# Difunctional Silicon Dioxide Combined with Graphene Oxide Nanocomposite to Enhance the Anticorrosion Performance of Epoxy Coatings

**DOI:** 10.1021/acsomega.2c00494

**Published:** 2022-07-01

**Authors:** Chun Feng, Lijuan Zhu, Kunyao Cao, Zongxue Yu, Yacong Song

**Affiliations:** † Tubular Goods Research Institute of China National Petroleum Corporation, Xi’an 710077, China; # State Key Laboratory for Performance and Structure Safety of Petroleum Tubular Goods and Equipment Materials, Xi’an 710077, China; ‡ Southwest Petroleum University, School of Chemistry and Chemical Engineering, Chengdu 610500, China; § Xi’an Shiyou University, School of Materials Science and Engineering, Xi’an 710065, China

## Abstract

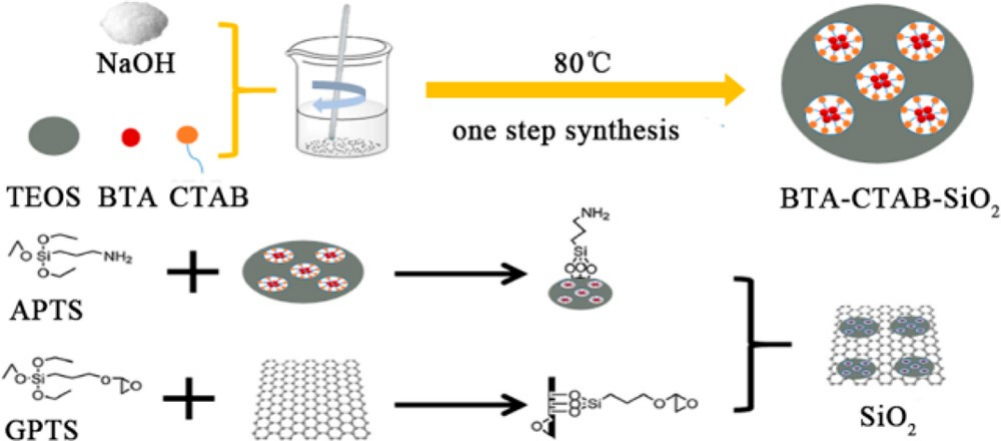

The nanocomposite BTA-SiO_2_-GO was fabricated for the purpose of metal corrosion protection. Herein, the BTA-loaded mesoporous silica nanocontainers were prepared through a facile one-step synthetic method. Subsequently, graphene oxide (GO) was combined with the resultant BTA-SiO_2_ compound because GO had a superior barrier property and impermeability. We must note that the double functional groups exist on SiO_2_. Benzotriazole (BTA), as an inhibitor, can be loaded into the nanocontainer and GO can also be modified by it, resulting in excellent dispersion in epoxy coatings, which were conducive to enhancing its anticorrosion performance. In this way, the nanocomposite endows the coating system with both self-healing and physical barrier abilities. The EIS results indicated that the impedance value of the BTA-SiO_2_-GO composite coatings was up to 1.2 × 10^9^ Ω cm^2^, which indicated excellent corrosion resistant properties.

## 1 Introduction

Conventional coatings have failed to keep up with the demands of the increasingly important problem of metal corrosion in modern industry and daily life.^1^ Intelligent coatings, which are generally composed of corrosion inhibitors and nanocontainers, are one of the most economical and widely used anticorrosion coatings, with a good pH response and self-healing ability.^2,3^ The corrosion resistance of coatings depends largely on the ability of the loading corrosion inhibitor and controlled release ability of the corrosion inhibitor.^4^ A variety of nanocontainers, including cyclodextrin, Eloite nanotubes, carbon nanotubes, metal–organic frameworks, zirconia, and mesoporous silica, have been reported, and mesoporous silica nanoparticles are a good example.^5^ The excellent compatibility, high specific surface area, and large pore diameter have the advantages of good dispersion and strong load capacity. Mesoporous silica nanoparticles have the ability to respond to pH, which is commonly referred to as controlling the release of inhibitors. Moreover, its mechanical stability in the same type of material is also quite excellent.^6,7^ It has been proven that a corrosion inhibitor can improve the corrosion resistance of coating through a number of studies. Nikpour et al. used *Urtica dioica* extract and GO to prepare anticorrosion coatings, but due to the complex and changeable corrosion environment, most of the extract would be released in advance, thus greatly reducing the anticorrosion efficiency.^8^ Benzotriazole (BTA) derivatives are one of the most effective and commonly used corrosion inhibitors for metals. Sheng et al. synthesized a synergistic nanocomposite using the oxidation reaction of aniline and GO, but this material can only be passively anticorrosive and shows a lack pH responsiveness.^9^ In this paper, mesoporous silica nanoparticles were synthesized to load BTA as smart coatings.^10−12^

Because the solvent in the curing process of the coating under high temperature conditions results in evaporation at the same time the coating produces pores or cracks, a corrosive medium will therefore penetrate into the metal matrix, thereby damaging the coating. Therefore, the single self-healing property is not enough to prevent metal corrosion.^13^ We prepared BTA-SiO_2_-GO nanocomposites by doping GO to improve the physical barrier properties of the coating because of GO’s excellent barrier and impermeability. However, the poor dispersion of GO in epoxy coating will greatly affect the corrosion resistance of the coating. The dispersion in epoxy coatings can be enhanced by surface modification of GO due to its rich oxygen-containing functional groups on the surface.^14−17^ It can be seen from the literature that the modified GO surface greatly improves the dispersion of the epoxy coating and thus improves the corrosion resistance of the coating. Cao et al. used ethyl orthosilicate to modify GO, which greatly improved the dispersion of GO in epoxy resin, thereby improving the corrosion resistance.^12^ We modified SiO_2_ with silane coupling agent (APTS) because SiO_2_ is an inorganic compound, which means it is difficult to bind tightly to oxygen-containing functional groups on the surface of GO. By grafting −NH_2_ onto SiO_2_ and the interaction between −NH_2_ and the epoxy group, modified SiO_2_ can be better grafted onto GO.^18−21^

**Scheme 1:**
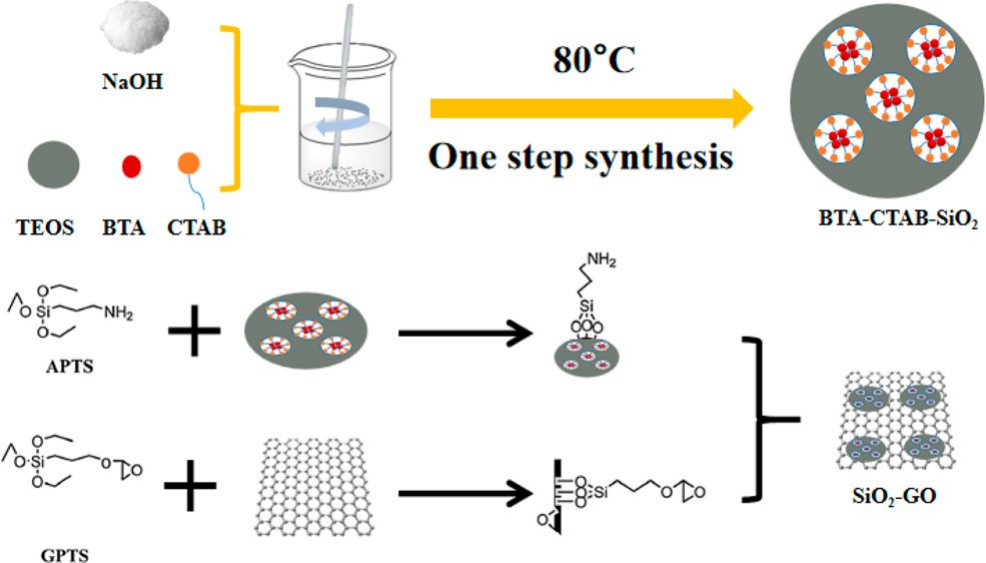
Illustration of the Procedure for Preparing BTA-SiO_2_-GO Nanocomposites

Even this does not result in the best corrosion resistance of the coating as there is uneven distribution of the epoxy groups on the surface of GO.^22−24^ Therefore, we modified GO using silane coupling agents (GPTS) to evenly distribute epoxy groups on the surface of GO. The results show that the prepared BTA-SiO_2_-GO has good dispersibility in epoxy coatings, and the coating system displays both corrosion inhibition and physical barrier properties. The first thing that comes into play is layered GO when the corrosive medium begins to penetrate the coating. It acts as a physical barrier by extending the path of the corrosive medium to the metal surface for delaying its arrival on the metal surface.^25−28^ Subsequently, BTA will be released from the mesoporous SiO_2_ nanovessel when the pH value changes due to electrochemical corrosion to adsorb on the corrosion site and produce a film to protect the metal matrix. In order to obtain BTA-SiO_2_-GO nanocomposites, the process shown in Scheme 1 is adopted.

## 2 Experimental Section

### 2.1 Materials

Graphite, phosphoric acid, sulfuric acid, sodium nitrate, hydrogen peroxide, potassium permanganate, tetraethyl orthosilicate (TEOS), benzotriazole (BTA), cetyltrimethylammonium bromide (CTAB), sodium hydroxide, 3-aminopropyltriethoxysilane (APTS), 3-glycidoxypropyltrimethoxysilane (GPTS), *N*,*N*-dimethylformamide (DMF), and ethanol came from Kelong Chemical Reagent Factory (Chengdu, China). The curing agent and epoxy resin (WSP-6101) were provided by Bluestar Technology WuXi Resin Factory. Deionized water was obtained from water purification equipment (UPC-III-40L, Ulupure). The carbon steels were polished using 800 grit sandpaper.

### 2.2 Preparation of the BTA-SiO_2_ Nanocontainer

The mesoporous BTA-SiO_2_ was synthesized by a facile one-step method. First, 0.21 g of sodium hydroxide was dissolved into 363 mL of deionized water at 80 °C. Subsequently, 0.75 g of cetyltrimethylammonium bromide (CTAB), 1.25 g of benzotriazole (BTA), and 3.75 g of tetraethyl orthosilicate (TEOS) were added, and then the resultant was stirred for 2 h at 80 °C. Subsequently, the resultant was centrifuged at a rate of 6000 w/min. Finally, the mixture was washed with ethanol three times followed by vacuum drying at 90 °C in the oven for 6 h. The BTA-SiO_2_ was fabricated.

### 2.3 Preparation of GO

GO nanosheets were prepared within the amended Hummer’s method.^13^ First, 100 mL of sulfuric acid (98%) and 1.8 g of graphite were put into an ice bath and stirred for 1.5 h. Then 8.8 g of KMnO_4_ was adjoined gradually to the resultant and stirred vigorously. Subsequently, the resultant was stirred for 24 h at room temperature. The mixture was watered down by addition of deionized water drop by drop, and then hydrogen peroxide was added to end the oxidation process. In the end, after centrifugation and washing by deionized water and vacuum drying at 60 °C for 24 h, the GO powder was prepared.

### 2.4 Modification of SiO_2_

A 0.1 g portion of SiO_2_ and 2 g of 3-aminopropyltriethoxysilane (APTS) were added to 90 g of ethanol, and ultrasonication was conducted. Then the resultant was stirred at 80 °C for 5 h. Meanwhile, 8 g of deionized water was slowly added. The resultant compound was centrifuged and washed (three times in pure water and three times in ethanol). Finally, the resultant was dried in a vacuum oven at 60 °C for 24 h.

### 2.5 Modification of GO

A 0.1 g portion of GO and 2 g of 3-glycidoxypropyltrimethoxysilane (GPTS) were added to 80 g of ethanol, and ultrasonication was conducted. After the mixture was stirred for 5 h at 80 °C, 8 g of deionized water was added. The mixture was centrifuged and washed (three times in pure water and three times in ethanol). In the last step, the obtained material was dried for 24 h in a 60 °C oven to obtain f-GO.

### 2.6 Preparation of BTA-SiO_2_-GO Composite

A 250 mg of f-GO was added to 100 mL of DMF, and ultrasonication was conducted for 15 min. Then 50 mg of f-SiO_2_ was added to the above solution, and ultrasonication was conducted for 15 min. The solution was stirred at 105 °C for 6 h. The resultant mixture was centrifuged and washed (three times in pure water and three times in ethanol).

### 2.7 Preparation of Composite Coatings

The coating was obtained by the following method. First, 5 mL of (10 mg/mL) of BTA-SiO_2_ aqueous was mixed with 1.8 g of curing agent. The compound was stirred for 30 min, followed by 30 min of ultrasonic treatment. In order to ensure that the BTA-SiO_2_ composite material has good dispersion in the coating, after the excess solvent was removed by centrifugation, 2 g of epoxy resin was added and the mixture stirred for 30 min. Subsequently, the mixture was put through the degassing step in a vacuum oven for 10 min at ambient temperature. Finally, the composite was coated with a brush to a thickness of about 100 μm on the pretreated carbon steel. The coated carbon steel was cured at room temperature for 48 h and then cured for 12 h in a 120 °C oven. The coating was named BTA-SiO_2_/EP. Pure epoxy coating and BTA-SiO_2_-GO/EP coating were prepared by using the above methods.

### 2.8 Instruments and Characterization

A WQF-520 infrared spectrometer (Beijing Rayleigh Analytical Instrument Co., Ltd.) was used for Fourier transform infrared spectroscopy analysis. The crystalline structures of these composites were investigated using an X-ray diffractometer (PANalytical, X’Pert PRO MPD) with a copper K α-radiation source. Thermogravimetric analysis (TGA) experiments were conducted to demonstrate the BTA was loaded into the nanocontainers successfully on a Q500 thermogravimetric analyzer (TA Instruments, USA). To evaluate the release behavior and ability of corrosion inhibitors, measurements were made using a UV–vis method on a Lambda 18 UV spectrometer. Porosity and surface area were tested using the Brunauer–Emmett–Teller (BET) method. The components of the samples were investigated through X-ray photoelectron spectroscopy (XPS, PHI 5000 VersaProbe). The structure of the composite materials was studied using a Raman test. The surface morphology was characterized by scanning electron microscopy (SEM, JSM-7500F, JEOL, Tokyo, Japan) images.

The electrochemical impedance spectroscopy (EIS) obtained from an electrochemical instrument (Shanghai Huachen Instrument Co., Ltd., China) was analyzed to study the corrosion resistance of the coating. In order to study the electrochemical behavior of different proportions of coatings in the traditional three-electrode system (experimental frequency range is 10^5^ to 10^–2^ Hz, sinusoidal disturbance amplitude is 10 mV), the electrical equivalent circuit of ZSimpWin software was used to record and fit the data.

## 3 Results and Discussion

### 3.1 Material Characterization and Assessment

#### 3.1.1 Infrared Spectrum (FTIR) Analyses

**Figure 1:**
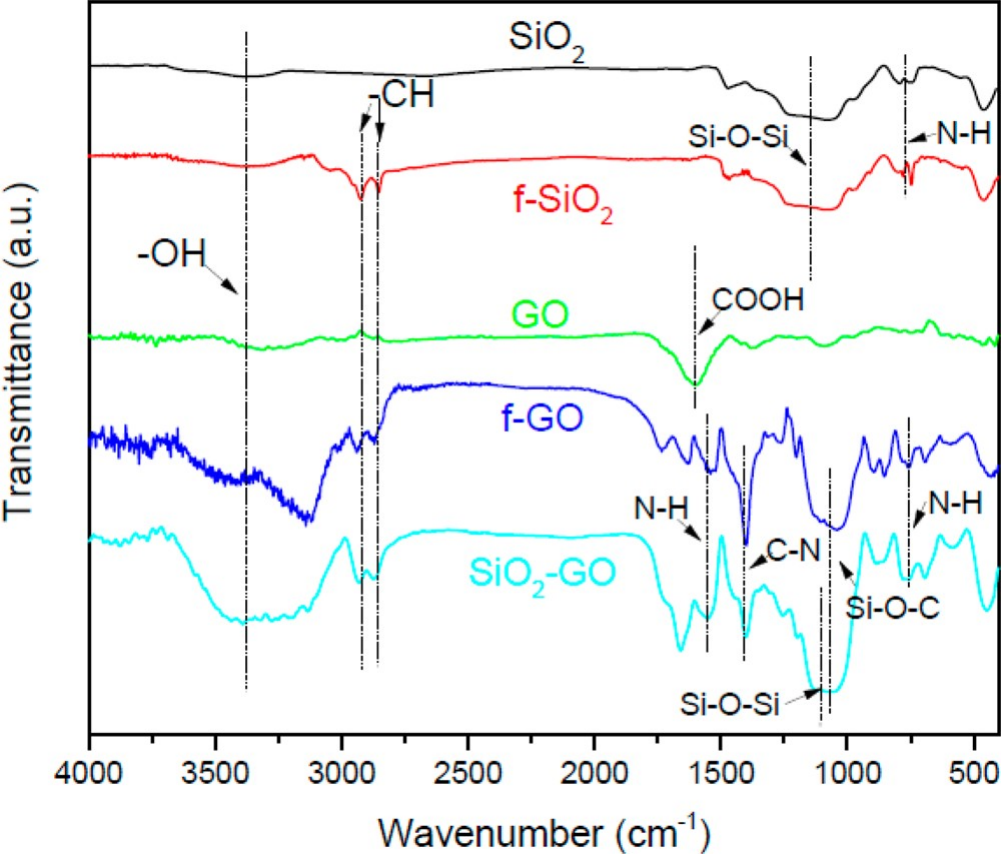
FTIR spectra of BTA-SiO_2_ and BTA-SiO_2_-GO.

Figure 1 shows the FTIR of SiO_2_, f-SiO_2_, GO, f-GO, and SiO_2_-GO. As for SiO_2_, a wide peak was detected at 3380 cm^–1^ which was due to the O–H stretching. Compared with SiO_2_, the C–H stretching peaks obtained at 2935 and 2878 cm^–1^ and the N–H peak detected at 814 cm^–1^ of f-SiO_2_ were attributed to the amendment of APTS. The peak at 1109 cm^–1^ represented Si–O–Si bonding. These results indicated the successful modification of SiO_2_ by APTS. The O–H tensile peak and −COOH characteristic peak on GO appeared at 3380 and 1590 cm^–1^, respectively. For f-GO, the C–H peaks at 2935 and 2878 cm^–1^ were presented. In addition, the weak peak at 1532 cm^–1^ was ascribed to the secondary amide N–H bending, and the N–H vibration peak could be observed at 814 cm^–1^ as well. Moreover, the C–N tensile peak and Si–O–C peak arose at 1456 and 1072 cm^–1^, respectively.^29−32^ These results vividly demonstrated that the GO was modified by GPTS and the nanocomposite SiO_2_-GO was prepared.

#### 3.1.2 X -ray Diffraction (XRD) Tests

**Figure 2:**
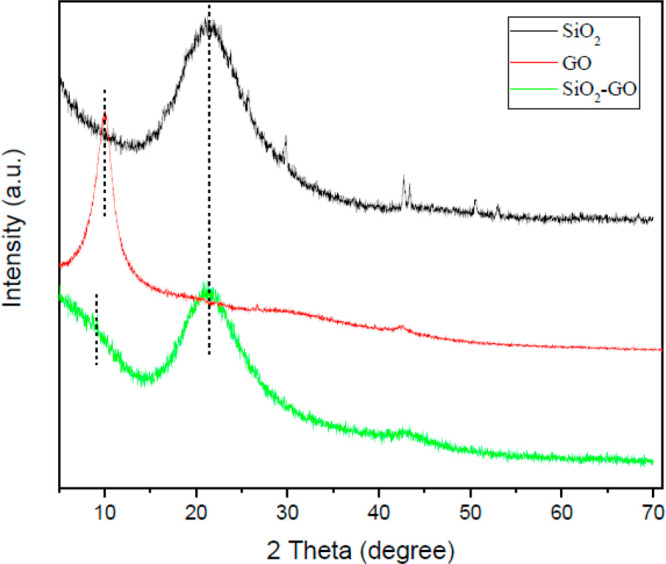
XRD pattern of BTA-SiO_2_ and BTA-SiO_2_-GO.

The crystal structures of SiO_2_, GO, and SiO_2_-GO were studied by wide-angle X-ray scattering, and the results are shown in Figure 2. The characteristic diffraction peak of SiO_2_ appears at 21°and is a wide peak, which proves that SiO_2_ has been successfully prepared. As for GO, the characteristic diffraction peak appeared at 9.8°.^33^ As for SiO_2_-GO, the broad peak at 21° could be observed as well. In addition, the GO characteristic diffraction peak at 9.8° almost disappeared. These observations demonstrated the successful combination between GO and SiO_2_.

#### 3.1.3 Thermostability Analyses

**Figure 3:**
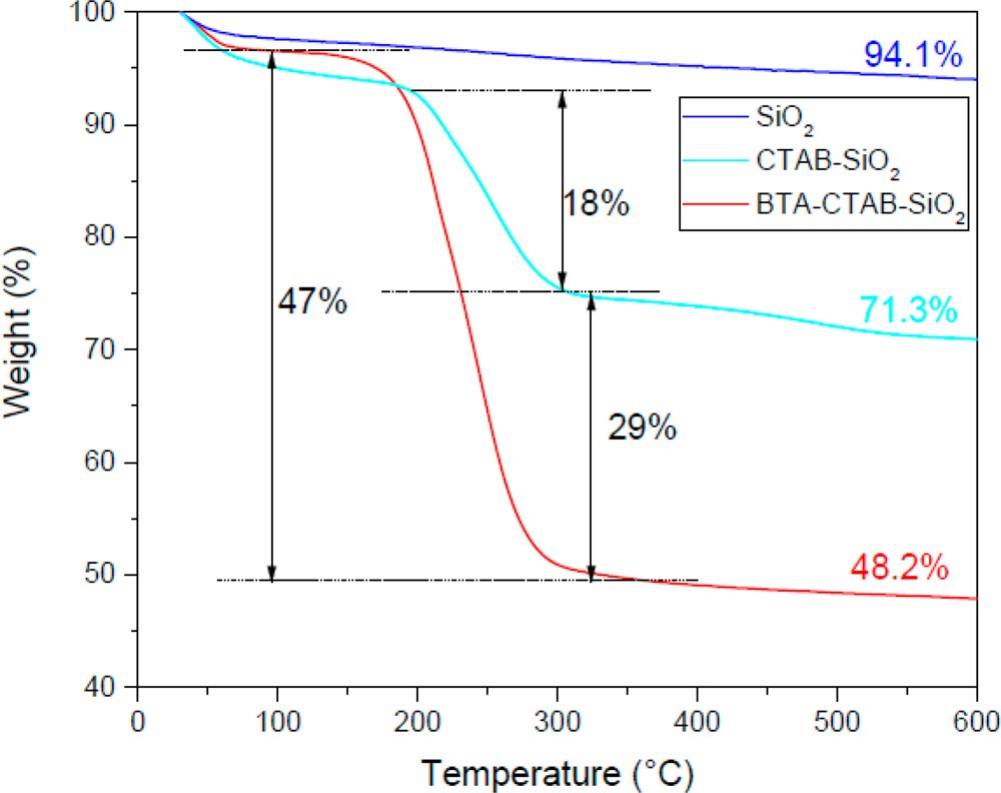
TGA curves of SiO_2_, CTAB-SiO_2_, and BTA-CTAB-SiO_2_.

TGA measurements were performed to estimate the BTA loading capacity of the nanocontainers. The weight losses for SiO_2_, CTAB-SiO_2_, and BTA-CTAB-SiO_2_ are displayed in Figure 3. In the first stage (below 100 °C), about 3% weight loss for three composites could be observed, which was due to the adsorbed moisture in powder. SiO_2_ has little weight loss, which proves SiO_2_ has good thermal stability. When CTAB is decomposed at 200–300 °C, the weight loss of CTAB-SiO_2_ is about 18%. As for BTA-CTAB-SiO_2_, about 47% weight loss can be observed at 100–300 °C; the first stage (begin at 100 °C) was ascribed to the decomposition of BTA, while the second stage (begin at 200 °C) was attributed to the decomposition of CTAB. According to the comparison, it is clear that the encapsulation amount of BTA was about 29%. From the above results, it can be concluded that SiO_2_ nanocontainers are successfully loaded with BTA corrosion inhibitor.

#### 3.1.4 Release of BTA Inhibitor

**Figure 4:**
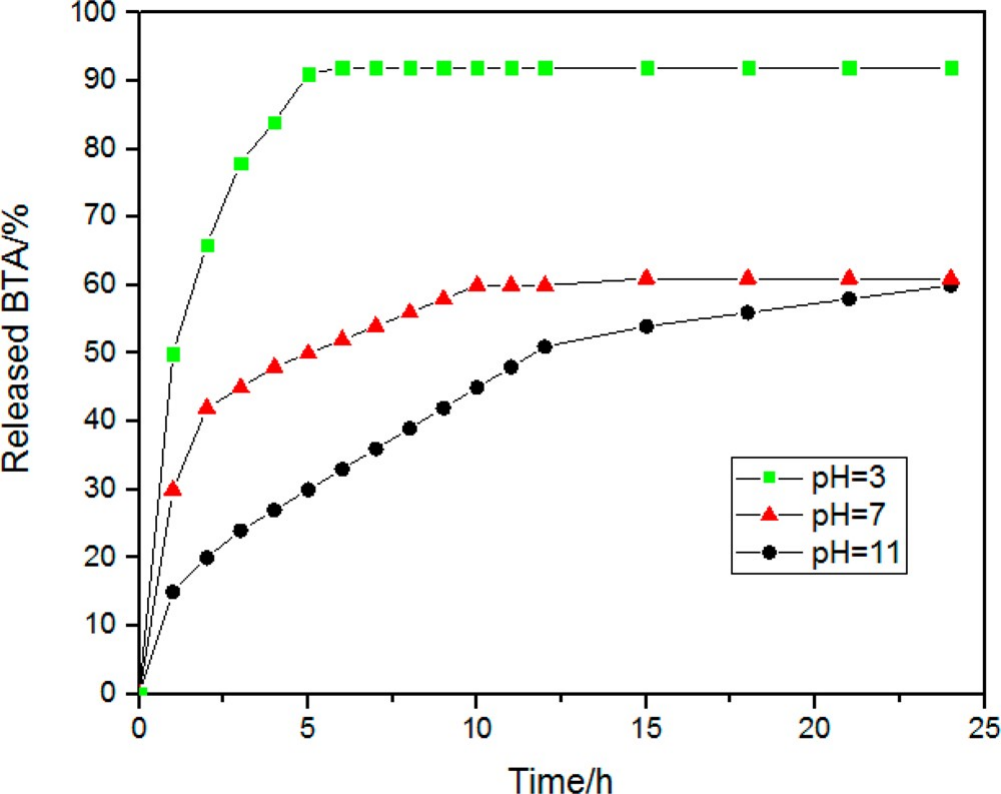
Profiles of BTA release from BTA-SiO_2_ at different pH values measured with UV spectroscopy.

The release of a corrosion inhibitor from the nanovessel was experimentally verified by UV–vis spectrophotometry. The absorbance intensity of BTA was recorded in 3.5 wt % NaCl suspension containing BTA-SiO_2_ with different conditions (pH = 3, 7, and 11). Figure 4 shows two stages of BTA release: rapid release and gradual release. Apparently, under different pH conditions, the release rate of BTA is different, indicating that the release progression is pH responsive. At pH 3, BTA was almost completely released for just 5 h, implying the speedy release rate and the hugest release amount. At pH 7 and 11, the initial stage lasted for 12 h, which was ascribed to a slow release rate. In addition, the release amount decreased a lot.

#### 3.1.5 BET Experiments

**Figure 5:**
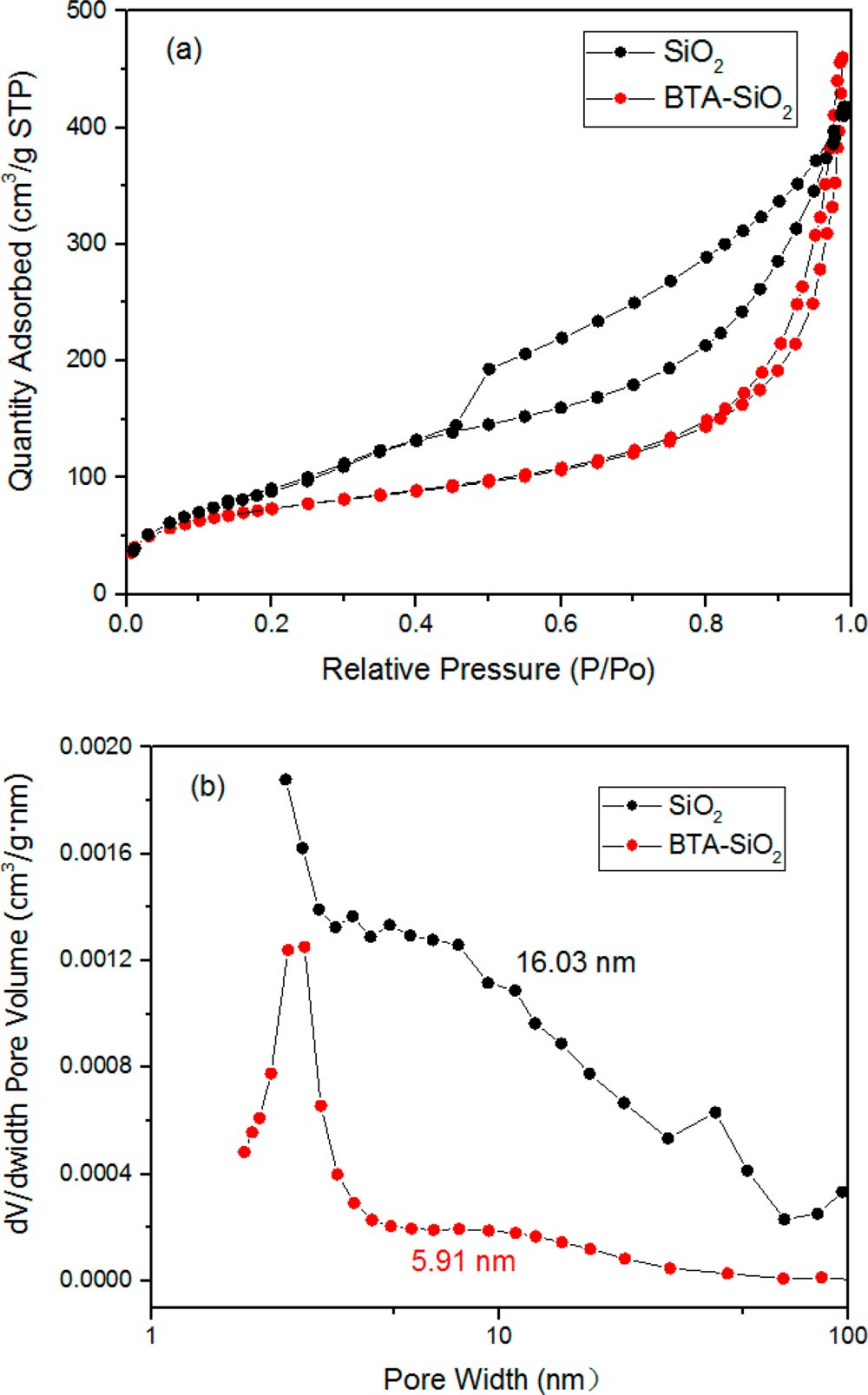
(a) Nitrogen adsorption/desorption isotherms for SiO_2_ and BTA-SiO_2_. (b) Pore size distributions of SiO_2_ and BTA-SiO_2_ based on the adsorption branch using the BJH algorithm.

As can be seen in Figure 5a, nitrogen adsorption/desorption results of SiO_2_ are of typical type IV (based on IUPAC definition) with a hysteresis loop in the P/P_0_ region of 0.45–0.95.^34^ This can be ascribed to the instability of liquid N_2_ in the narrow channels, indicating the presence of a mesoporous material, confirming the successful fabrication of mesoporous SiO_2_. As for BTA-SiO_2_, the hysteresis loop disappeared, which indicated that the loading of BTA and CTAB nanoparticles occupied the mesoporous material, confirming the successful loading of BTA into SiO_2_. Also, this can be proved by the pore size distribution of the two samples. As can be seen from Figure 5b, the average pore size of SiO_2_ was about 16.03 nm. After the loading of BTA, the average pore size was decreased to 5.91 nm. These results proved the prosperous loading of BTA into SiO_2_.

#### 3.1.6 XPS Analyses

**Figure 6:**
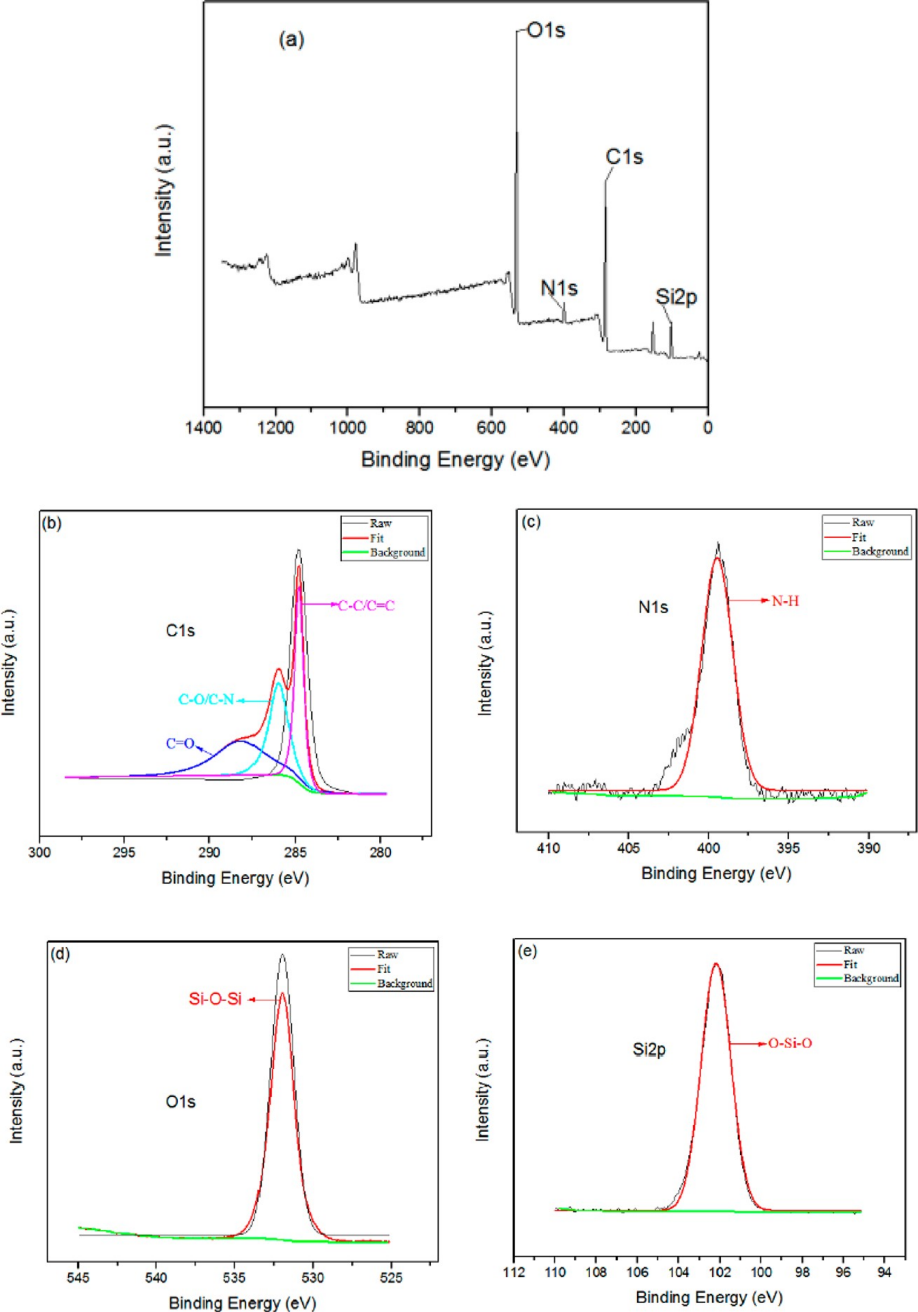
High-resolution XPS spectra of BTA-SiO_2_-GO: (a) full survey spectrum; (b) C 1s, (c) N 1s, (d) O 1s, (e) Si 2p.

The composition of BTA-SiO_2_-GO nanocomposites can be expressed by XPS tests, and these results are presented in Figure 6. It can be seen from Figure 6a that C, N, O, and Si elements exist in the composite material. Apparently, in Figure 6d, the peak of the Si–O–Si binding energy is 531.98 eV, and in Figure 6e, the peak of the O–Si–O binding energy is about 102.17 eV, indicating the successful synthesis of SiO_2_.^35^ In addition, the peaks of binding energies of 284.81, 286.06, 288.32, and 399.55 eV represent C–C/C=C, C–O/C–N, C=O, and N–H, respectively, while the C–C/C=C, C–O/C=O were ascribed to the GO and the silane coupling agent (APTS/GPTS) and the −N–H was ascribed to the amido bond of the combination of f-SiO_2_ and f-GO.^36^ These results showed that the BTA-SiO_2_-GO nanocomposite was fabricated successfully.

#### 3.1.7 Raman Test

**Figure 7:**
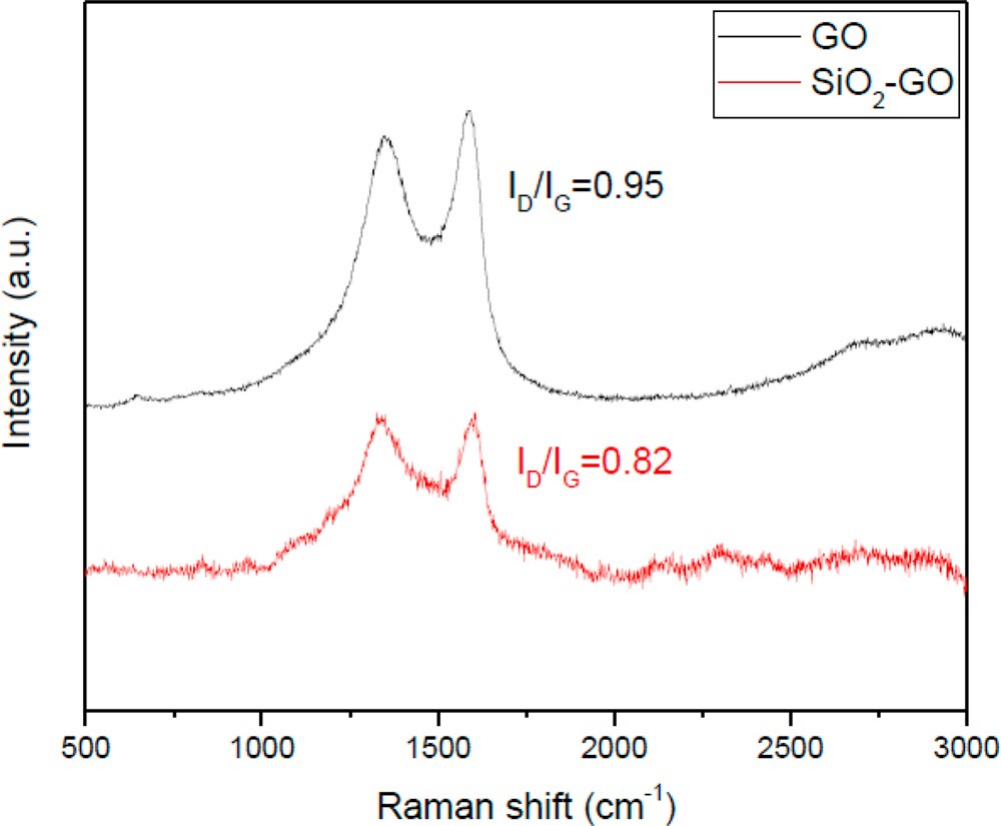
Raman spectra of GO and SiO_2_-GO.

Figure 7 displayed the Raman spectra of GO and SiO_2_-GO. As expected, the samples displayed the typical lower intensity D band at 1360 cm^–1^ along with a G band at 1595 cm^–1^.^37^ The D band represents a defect in the lattice of carbon atoms, and the G band represents sp^2^ hybridization of carbon atoms for in-plane stretching vibration. The *I*_D_/*I*_G_ ratio of GO was 0.95, while the *I*_D_/*I*_G_ ratio of SiO_2_-GO was 0.82. The decrease demonstrated that the SiO_2_ particles had replaced the carbon atoms and anchored onto the GO surface, indicating the successful combination of SiO_2_ and GO.

#### 3.1.8 Morphological Studies

**Figure 8:**
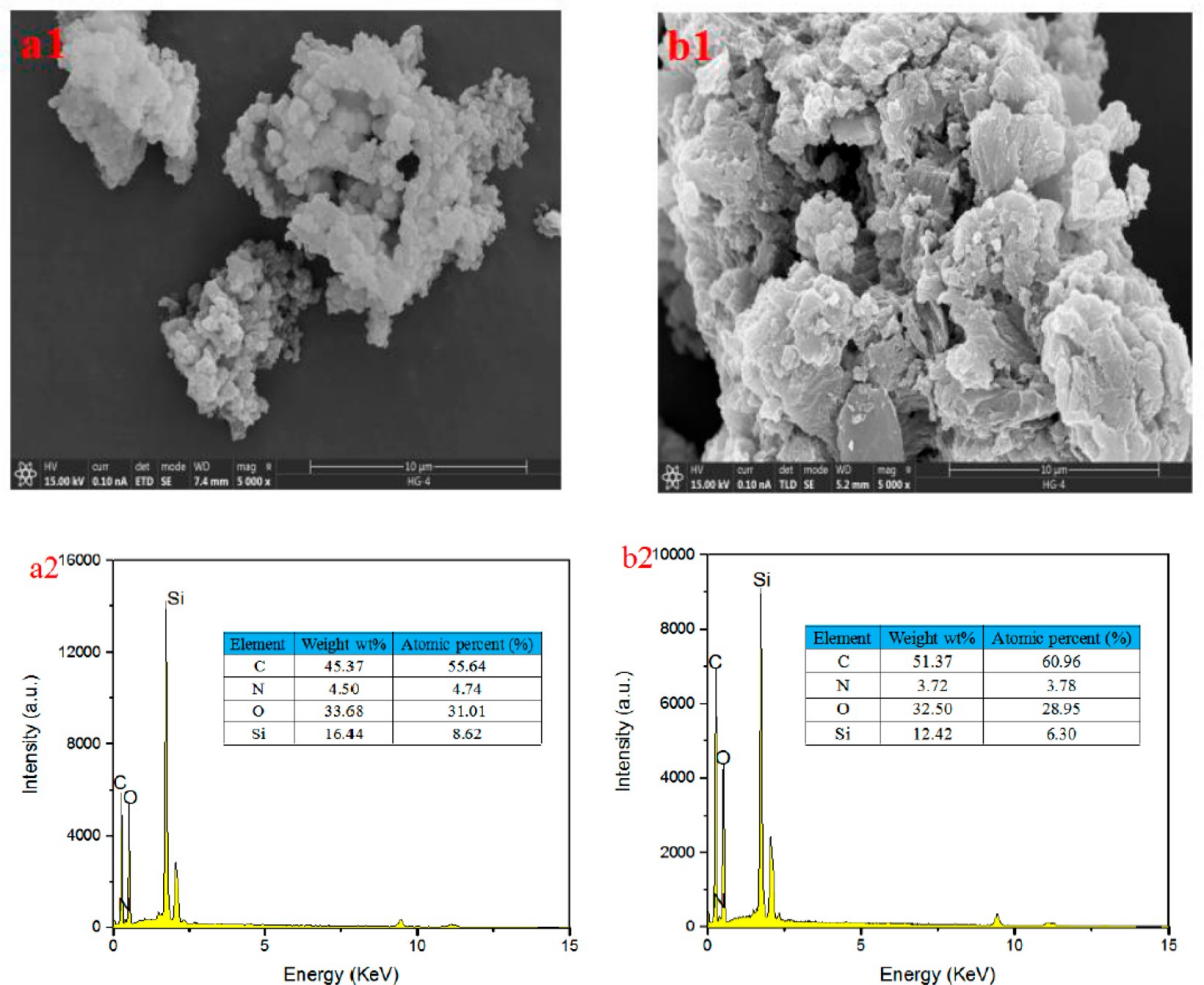
Scanning electron micrograph and energy spectrum of (a) BTA-SiO_2_ and (b) BTA-SiO_2_-GO.

SEM analyses were carried out to explore the morphology of BTA-SiO_2_ and BTA-SiO_2_-GO. Figure 8a1 displays the morphology of BTA-SiO_2_, which are spherical particles in different shapes. As shown in Figure 8b1, the BTA-SiO_2_-GO particles show a smaller size and better dispersibility. Additionally, it can be observed that the BTA-SiO_2_ particles were loaded on GO. Moreover, the mapping of EDS analysis proved the presence of N and Si elements, proving the merging of BTA and SiO_2_. These consequences showed that two nanocomposites were made up well.

### 3.2 Characterization of Coatings

#### 3.2.1 Section Results of Coating

Scanning electron microscopy (SEM) was used to observe the fractured surface of the coating to verify the compatibility and dispersity between the prepared nanocomposites and the resin. Surfaces without added composites are smooth and shiny. However, there were some micropores (red circle). As for GO/EP coating, obvious agglomeration and layers stacked could be observed, which was attributed to the strong van der Waals forces. There were a lot of aggregations in BTA-SiO_2_/EP coating, implying the weak dispersion. As for BTA-SiO_2_-GO/EP coating, the aggregation phenomenon was apparently reduced, which was attributed to the modification of SiO_2_ to GO, resulting in the excellent compatibility between BTA-SiO_2_-GO and the epoxy resin.

#### 3.3.1 Electrochemical Impedance Spectroscopy Analyses

In general, the corrosion process of a coating is always composed of the following two stages. Usually, an EIS measurement method is used to test and evaluate the anticorrosion performance of nanocomposite coatings with different proportions under different immersion time. EIS measurement results and equivalent circuits are shown in Figure 10. In the first stage, the corrosive substance moves away from the metal matrix and is represented by a time constant. In the second period, the corrosive material has entered the coating and penetrates the coating into contact with metal matrix and begins the corrosion progression, communicated by the second time constants.^38−41^

**Figure 9:**
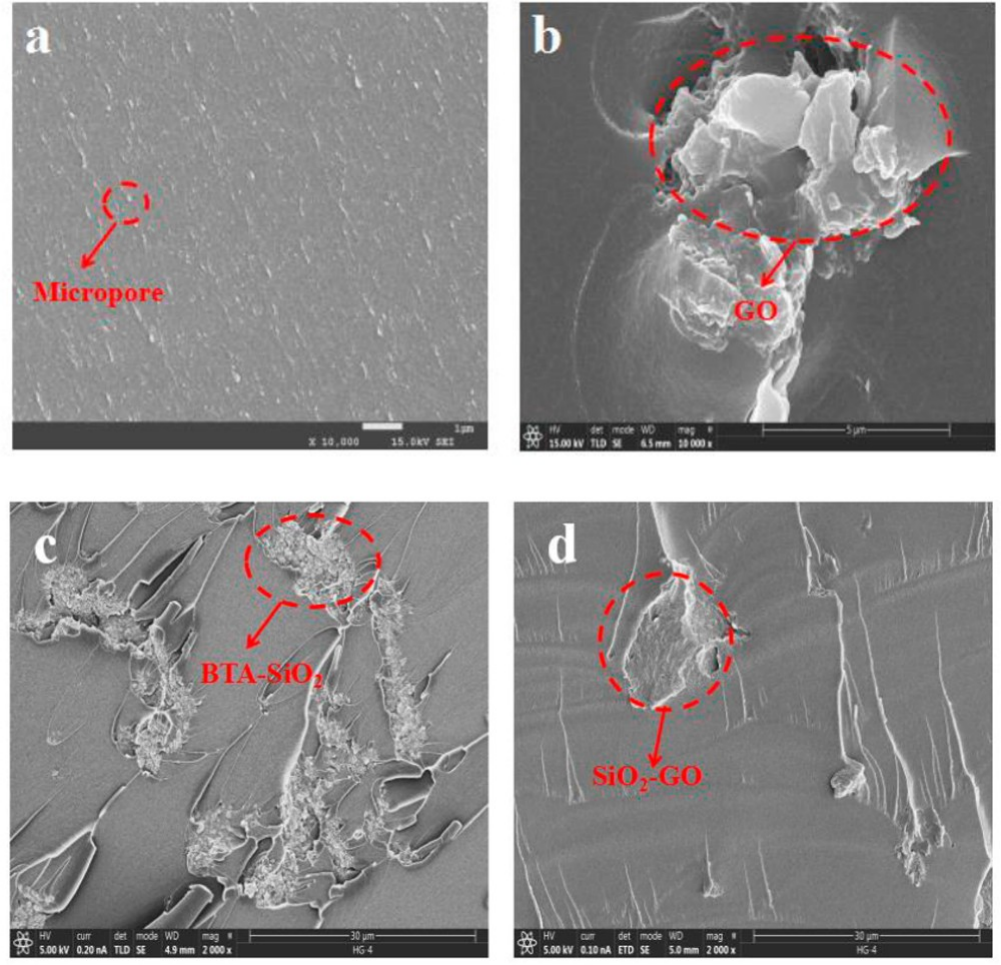
Fracture surfaces of (a) pure EP, (b) GO/EP, (c) BTA-SiO_2_/EP, and (d) BTA-SiO_2_-GO/EP.

**Figure 10:**
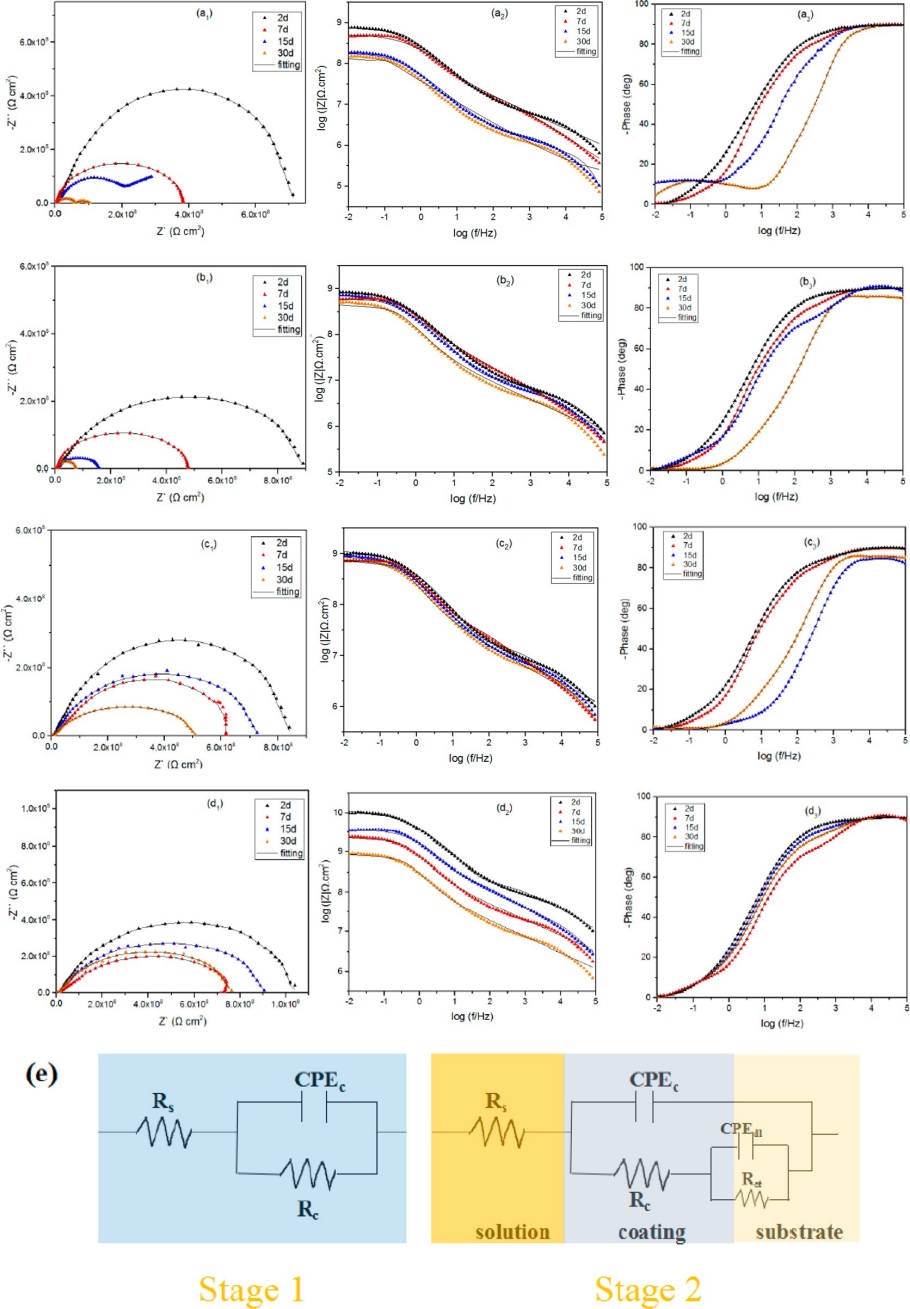
Bode and Nyquist plots of (a) pure EP, (b) GO/EP, (c) BTA-SiO_2_/EP, and (d) BTA-SiO_2_-GO/EP coatings at different immersion times in 3.5 wt % NaCl solution. (e) The corresponding equivalent electric circuit.

Nyquist plots are shown in Figure 10a. The impedance modulus of pure EP coating decreased dramatically from 7.12 × 10^8^ to 3.81 × 10^8^ Ω cm^2^ after a 7 day immersion process. The impedance modulus decreased to 2.0 × 10^8^ Ω cm^2^ after 15 days of immersion and further decreased to 5 × 10^7^ Ω cm^2^ after 30 days. The appearance of two impedance arcs means that the time constant becomes two. The pure EP coating has micropores or microcracks, resulting in poor barrier performance (Figure 9a). The GO/EP coating accelerates the corrosion behavior of the matrix. When GO is added into the epoxy resin, the agglomeration phenomenon (Figure 9b) results in poor dispersion. EIS measurements showed that the impedance decreased faster than pure EP coating. In contrast, the impedance of the BTA-SiO_2_/EP coating is 8.3 × 10^8^ Ω cm^2^ at the initial immersion stage. Due to the active inhibition properties of the BTA-SiO_2_/EP coating, the impedance of coating increased significantly at 15 days. Meanwhile, the slow decline of impedance modulus of the coating can also be attributed to its active inhibition performance. The impedance modulus of BTA-SiO_2_-GO/EP coating is further increased to 1.3 × 10^9^ Ω cm^2^ at the beginning of the soak. GO has a physical barrier, and BTA-SiO_2_-GO nanocomposites are evenly dispersed in epoxy coating. In addition, the active suppression performance of the coating is better reflected in that the impedance modulus of the 15th and 30th day is higher than that at the seventh day.

In addition, it is shown from the Bode diagram (Figure 10b) that the change of impedance modulus of EP coating at *Z*_f_ = 0.01 Hz is related to the change of immersion time. Different from pure EP coating, the impedance of BTA-SiO_2_/EP and BTA-SiO_2_-GO/EP coatings rebound in numerical value, which is a manifestation of the proactive inhibition presentation of BTA- SiO_2_/EP coatings. According to the bode phase angle diagram of the pure EP coating (Figure 10c), the maximum value of the low-frequency region indicates that the barrier has weak anticorrosion presentation, which is consistent with the results of Figure 10a1. The change of breakpoint frequency can reflect the coating inhibiting the diffusion of corrosive media. Among the increase of immersion time, the breakpoint frequency (*F*_b_) increases, which proves that the corrosive medium has spread to the surface of the metal matrix. With the extension of immersion time, the phase Angle values of BTA-SiO_2_ /EP coating and BTA- SiO_2_-GO/EP coating at 10 kHz show a difference. Compared with the coating containing GO, the coating without GO shows a downward trend, and the breakpoint frequency also increases. This may be due to the difference caused by GO outstanding barrier properties. In summary, the corrosion resistance of the prepared BTA-SiO_2_-GO/EP coating can be ascribed to the following reasons: Corrosion inhibition ability of BTA, active release of corrosion inhibitor by SiO_2_, and strong physical barrier of GO.

#### 3.3.2 Electrochemical Analysis under Acidic Conditions

**Figure 11:**
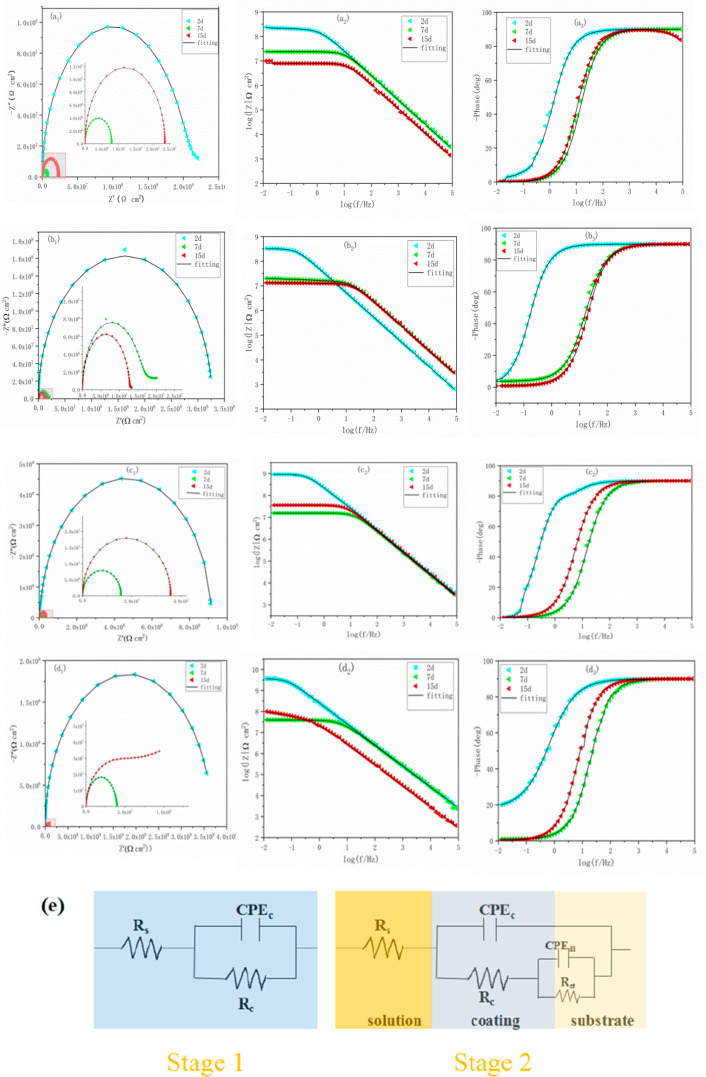
Bode and Nyquist plots of (a) pure EP, (b) GO/EP, (c) BTA-SiO_2_/EP, and (d) BTA-SiO_2_-GO/EP coatings at different immersion times under acidic conditions (pH = 3). (e) Corresponding equivalent electric circuit.

In order to ensure the pH of the solution (pH = 3), HCl is added to the 3.5 wt % NaCl solution. As shown in the Nyquist diagram (Figure 11a), for the EP coating, the impedance modulus cut severely from 2.21 × 10^8^ to 2.57 × 10^8^ Ω cm^2^ after 7 days immersion in acid solution (pH = 3). After 15 days of soaking, the impedance was reduced to 8.74 × 10^6^ Ω cm^2^. This result is caused by the micropores and cracks in the pure EP coating itself. An acidic environment will accelerate corrosion. In pH = 3 solution, the GO/EP coating appears to have worse corrosion resistance, which is reflected in the impedance spectrum as the impedance modulus decreases more rapidly (from 3.25 × 10^8^ to 2.02 × 10^7^ Ω cm^2^) than the EP coating behind soaking for the equal time. Because of GO agglomeration, the corrosion of the metal matrix was accelerated. In addition, an acidic environment will accelerate corrosion. For BTA-SiO_2_/EP coating, the initial value of impedance modulus reaches 9.13 × 10^8^ Ω cm^2^, and the impedance modulus decreases slowly with the extension of immersion time. The active release of BTA in the BTA-SiO_2_/EP coating made the impedance on the 15th day better than the 7th day. The impedance of BTA-SiO_2_-GO/EP coating is further enlarged to 3.61 × 10^9^ Ω cm^2^ at the initial stage of soaking in acidic solution of pH = 3. This result is caused by the combined effect of the barrier of GO to corrosive media and the better dispersion of BTA-SiO_2_-GO composite nanomaterials resin. The impedance of BTA-SiO_2_/EP and BTA-SiO_2_-GO/EP after immersion for 7 days decreases slower than that of pure EP and GO/EP due to the increase in the release rate of BTA under acidic conditions.

The impedance of pure EP weakens with the delay of soaking time near the ground frequency (*Z*_f_ = 0.01 Hz) of the bode-frequency diagram (Figure 11b). The impedance modulus rebound of the coatings containing BTA was observed, indicating that BTA can inhibit corrosion and increase the release rate under acidic conditions. Above and beyond, the change of the breakpoint frequency (*f*_b_) and the phase angle values again proves that the hurdle of coating is not adequate to keep the corrosive medium away from the metal substrate. The breakpoint frequency of BTA-SiO_2_-GO/EP composite coating hardly changes, which could be owed to the marvelous barrier property of GO. In summary, BTA-SiO_2_-GO/EP coatings show brilliant corrosion resistance. This is ascribed to the amazing corrosion inhibition property in acid solution (pH = 3) and the superior control release ability of SiO_2_ and admirable physical obstacle presentation of GO.

### 3.4 Schematic of the Active Inhibition Mechanism

**Figure 12:**
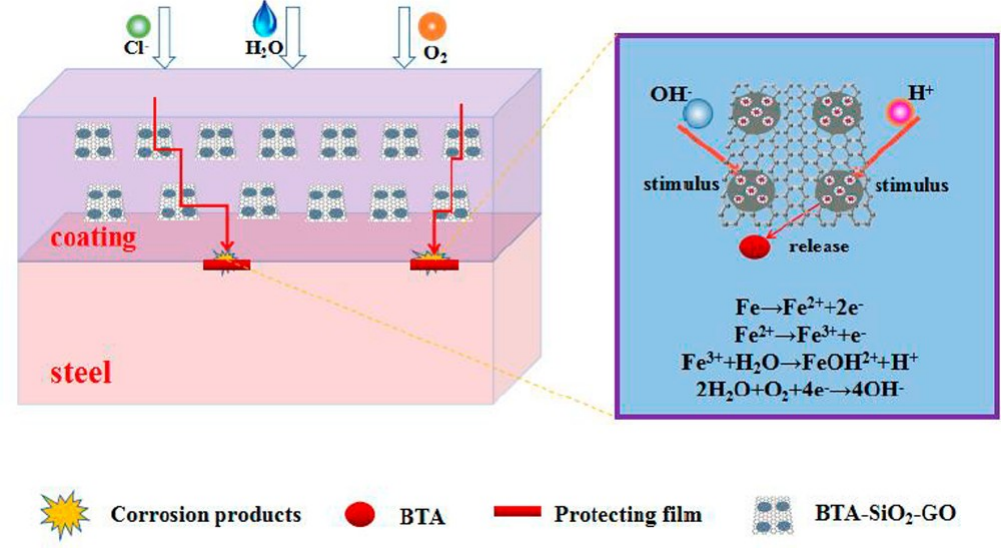
Schematic representation of the active inhibition mechanism for BTA-SiO_2_-GO/EP coatings.

It is known that the curing process can initiate micropores and cracks in the coating, which is ascribed to the solvent’s evaporation. It is because of their presence that the corrosive medium is able to contact the metal matrix and cause corrosion. Under these conditions, the homodispersion of BTA-SiO_2_-GO will become a physical barrier in restricting the corrosive mediums penetrating into the metal substrate at first, which greatly prolongs the corrosion path and the corrosion time. More importantly, metal corrosion is always accompanied by electrochemical corrosion, which will cause the pH value change. While the pH value changes, the BTA molecules can release from SiO_2_ nanocontainers and adsorb on the corrosion area to put their corrosion inhibition property to good use. The illustration of the active inhibition mechanism is shown as Figure 12, which vividly reveals the outstanding barrier property and impermeability of GO and the active inhibition of BTA.

## 4 Conclusions

In summary, the fabrication of the BTA-SiO_2_-GO nanocomposite has greatly enhanced the anticorrosion performances of epoxy coatings. While the SiO_2_ is difunctional, it has the ability to load BTA and modify the dispersibility of GO in the epoxy coatings, endowing the coating system with a uniform distribution, resulting in the enhancement of anticorrosion performances of epoxy coatings. In addition, the superior physical barrier performance and impermeability of GO improves the durable corrosion resistant property of epoxy coatings. Moreover, the active inhibition of BTA released from SiO_2_ nanocontainers plays a corrosion inhibition role in protecting metal matrix. Accordingly, the BTA-SiO_2_-GO/EP coating not only shows the durable corrosion resistance property but also shows the excellent active inhibition performance. Overall, the nanocomposite coating fabricated in the work shows enormous potential for applications for metal corrosion protection.
